# Impact of urban and rural residents medical insurance on self-rated health of residents in China: a panel study from the China family panel studies national baseline survey

**DOI:** 10.3389/fpubh.2024.1349416

**Published:** 2024-07-04

**Authors:** Yu Si-Yuan, Chen Ya-Ting, Xiao Xiao-Yue, Wu Dan, Lin Xin-hao, Liu Wen, Pei Tong, Meng Xue-Hui

**Affiliations:** School of Humanities and Management of Zhejiang Chinese Medical University, Hangzhou, China

**Keywords:** urban and rural resident medical insurance, health performance, resident, selfrated health, propensity score matching and difference-in-difference regression

## Abstract

**Objective:**

This study aimed to investigate the health performance of the Urban and Rural Residents Medical Insurance (URRMI) scheme in China and to make practical recommendations and scientific references for its full implementation in China.

**Methods:**

This is a panel study that uses data from the China Family Panel Studies from 2018 to 2020, which is separated into treated and control groups each year, utilizing the key approach of propensity score matching and difference-in-difference (PSM-DID). Using 1-to-1 k-nearest neighbor matching, we proportionate the baseline data. Using difference-in-difference model, we examine the mean treatment impact of the outcome variables. Using a 500-time random sample regression model, we validate the robustness of the model estimation.

**Results:**

The result was credible after matching, minimizing discrepancies. Good overall performance of self-rated health with an average Hukou status of, respectively, 0.8 and 0.4 in the treated and control group, primarily in rural and urban regions separately. The participation of URRMI significantly impacted self-rated health of residents, with a 0.456-unit improvement probabilities observed (*p* < 0.1). Additionally, the individuals are categorized into urban and rural, and those with urban hukou had a 0.311 expansion in the probability of having better health status compared to rural hukou (*p* < 0.05). Other factors, such as age, highest education, annual income, medical expenditure, hospital scale, clinic satisfaction, and napping, also impacted self-rated health. Moreover, elder individuals, higher education levels, and higher medical expenditure having a higher probability of improvement. The study utilized a placebo test to verify the robustness of the URRMI regression. The estimated coefficients showed that basic medical insurance did not significantly improve the health of insured residents under the URRMI scheme.

**Conclusion:**

The study demonstrates the crucial role of PSM-DID in determining the influence of URRMI on self-rated health status. It indicates that purchasing in URRMI has a favorable influence on the health of residents, advancing enhanced self-rated health effectiveness. It does, however, reveal geographical disparities in health, with urban dwellers faring far better than those who live in the suburb. Study suggests expanding URRMI coverage, narrowing urban–rural divide, increasing insurance subsidies, reforming laws, and developing effective advertising strategies.

## 1 Introduction

The World Health Organization (WHO) implemented Universal Health Coverage (UHC) in 2015, which ensures that populaces have admittance to the health care they require exclusive of incurring financial difficulty. UHC is one of the sustainable development goals (SDGs) approved by the world’s states in 2015. The WHO also proposed the UHC2030 plan, which is a global campaign to establish strong health systems to achieve UHC. The approach to Health Systems Strengthening (HSS) is crucial for achieving UHC. The healthcare system is generally understood as all authorized public and private organizations, institutions, and resources that are responsible for improving, maintaining, and restoring health. HSS entails investment in participation in an incorporated and systemic approach and altering the architecture that governs how various components of the health system operate and interrelate to address primary health needs through people-centered amalgamated services (1). As a result, HSS is the primary means of achieving UHC.

The Chinese government has always prioritized the health of its citizens, demonstrating a commitment to improving the accessibility to healthcare and affordability of medical costs for all in its programs. A paramount goal of the government is to achieve UHC of the basic social medical insurance system and to relieve the financial burden associated with medical treatment.

The basic social medical insurance system in China comprises the NRCMS in rural areas and the UEBMI and URBMI in urban areas. The basic social medical insurance system in China comprises the New Rural Cooperative Medical Scheme (NRCMS) in rural areas, and the Urban Employee Basic Medical Insurance (UEBMI) and Urban Resident Basic Medical Insurance (URBMI) in urban areas. In order to better solve the problem of medical resource imbalance caused by the urban–rural dual structure in China, the Central Committee of the Communist Party of China proposed the integration of the Urban and Rural Residents Medical Insurance System (URRMI) in November 2013, which was officially implemented in 2016.

With the swift economic expansion and appropriate improvement of medical and health facilities in China, the social security system has gradually begun to detach from construction, imposing a severe financial burden on the masses seeking medical treatment. By 2000, the proportion of personal health expenditure borne by Chinese residents had peaked, surpassing 60.6% of the overall health expenditure in China (2), and illness-related poverty had become a leading driver of poverty. Since the late 1990s, medical and health system reform has focused on constructing and improving a basic social medical insurance system. In 2013, China achieved complete coverage of the basic medical security system. By the end of 2021, the number of Chinese dwellers on national basic medical insurance was 1362.97 million, with a participation percentage of more than 95% (3). The proportion of China’s total health expenditure directly born by individuals and households has decreased annually, from 60% in 2001 to 27.7% in 2021 (4), with improvements in population coverage and security. Additionally, the problematic of “kan bing nan, kan bing gui,” which means the difficulty and high cost of getting medical treatment, has been significantly alleviated.

The UEBMI system was first designed to advance the coverage of urban medical insurance in China. It is a social security scheme designed to compensate urban employees and pensioners but not their families for economic losses caused by disease risk, introduced in two medium-sized cities (Zhenjiang and Jiujiang) in 1994 and expanded countrywide in 1998 (5). It went into effect in 1998 to cover urban employees, while the URBMI was implemented in 2007 to cover urban inhabitants (6). The UEBMI benefit packages are envisioned to cover not just inpatient medical care but also outpatient facilities, such as medical treatment for serious and chronic diseases (7). By the end of 2021, the number of employees enrolled in medical insurance was 354.31 million, a 9.76 million rise and a 2.8% increase over the previous year (3).

The NRCMS is a government-led voluntary insurance system established in 2003 to increase access to health insurance for rural citizens (8). Unlike obligatory insurance, the NRCMS is run and administered by the county. The central government connected the delivery of its subsidies to the extent of coverage in each country, providing local governments with a strong incentive to expand coverage. Enrollment in the NRCMS is typically centered on households rather than individuals, which is one of the most effective strategies for quick coverage expansion (9). The URBMI was launched with significant government subsidies and is designed similarly to the NRCMS. Its primary beneficiaries are children, the older adult, college students, and unemployed urban dwellers who are not covered by the UEBMI plan (10). After the implementation of the basic medical insurance system for urban employees and the new rural cooperative medical care system, it is a major initiative taken by the CPC Central Committee and the State Council to further address the healthcare security issues of the broad masses of people and continuously improve the healthcare security system. It mainly makes institutional arrangements for non-employed urban residents’ medical insurance. The introduction of this system is of great significance in the process of China’s social insurance system reform, indicating the direction of China’s social insurance system reform.

Following the incorporation of the URBMI into the NRCMS in 2016, China has founded a basic social medical insurance system, with the main entities being the UEBMI and the URRMI schemes. The merger of both schemes has improved the justice of the basic medical insurance system. By enrolling in a cohesive medical insurance system for urban and rural residents, residents can more fairly enjoy basic medical security rights and interests in accordance with the unified policy of insurance payment and treatment (11). The insurance benefits became more balanced following the merger and integration. Anyone with a recognized permanent residence in the countryside can be treated the same as those in cities. This equitable approach makes medical insurance reimbursement more practical and enables the basic medical “service packages” that the general public can access to be upgraded to meet higher standards. The basic medical “service packages” include expanding medical insurance reimbursement and broadening the variety of drugs enclosed in medical insurance. The use of URRMI enables the implementation of integrated handling service management, making management more unified. It removes barriers such as urban–rural system separation, management segmentation, and resource dispersion, making the transfer and linkage of medical insurance relationships amid urban and rural inhabitants more convenient.

Due to differences in healthcare insurance systems across countries, such as the commercial healthcare insurance system in America, the national health service system in Britain, and the social medical insurance system in China, it can still be found that healthcare insurance can provide certain assistance to the health of policyholders. Shi et al. from America investigated the association among insurance rank and general survival in female breast cancer patients attending public hospitals and observed that uninsured individuals outlive insured patients (12). Using data from groups in U.S. states, Thornton et al. observed that participation in commercial health insurance could save more than 75,000 lives per year by improving population health outcomes while extending commercial health insurance coverage to all uninsured people in the United States (13). A study showed that after 1 year of enrollment, enlarging health insurance had momentous effects on self-reported, mental, and physical health, although there was no substantial effect on humanity during the observation period (14). Another study using nationwide illustrative statistics from the Demographic and Health Surveys of Ghana, Indonesia, and Rwanda found that expanding health insurance to involve income-sensitive extras or exemptions for people with low income, as well as low or no copayments, can surge the usage of affectionate health care (15).

Chinese researchers also did numerous empirical research on health performance, exclusively concentrating on basic medical insurance. Corresponding to a study that used figures from the China Health and Nutrition Survey, participation in the new agricultural cooperation reduced the pervasiveness of some diseases, increased the pervasiveness of injuries and various diseases, and significantly improved participants’ self-rated health status (16). Shanshan et al., using this data analogously, observed that the new rural cooperative and urban housing insurance had no obvious positive effect on children’s short-term health, but the new agricultural cooperative had a meaningful constructive effect on children’s long-term health and significantly improved their health status (17). Based on data from the two phases of the China Health and Retirement Longitudinal Study, Lianjie et al. determined that integrating medical insurance for urban and rural inhabitants promotes the physical and mental health of older adult individuals in rural regions by increasing medical service consumption (18). Another study evaluated the temporary and continuing health evaluation indicators and found that the basic medical insurance of urban employees increased the actual medical costs of the insured population (19). However, it also had an optimistic effect on the health of the insured population or produced positive health performance (19). Meng et al. used statistics from the 2015 China Migrants Dynamic Survey to study the senior floating population and found that involvement in the health insurance system considerably improved the self-rated health of floating seniors (20).

Studies on the quantitative evaluation of medical insurance have some limitations. First, most studies rely on prevalence and mortality as evidence of the health effects of insurance, and there is less discussion about the causal link between self-rated health status and registration in insurance. Second, uncertainties persist concerning the effect of medical insurance on health, with some studies demonstrating no significant positive effect. Finally, most analyses have focused on the positive health promotion effect of health insurance, but the researches on URRMI insurance are limited. According to the health production theory of health economics, numerous factors influence health, including genetic inheritance, healthy behavior, dietary status, living environment, and medical services. The connection concerning health insurance and health is complicated because, under the idea of voluntary insurance, insurance status is frequently the product of human choice and an endogenous variable. Therefore, identifying the causative connection concerning health insurance and health status voguish studies is challenging.

This study used data from the large-scale China Family Panel Studies (CFPS) from 2018 to 2020 and propensity score matching and difference-in-difference regression (PSM-DID) to evaluate the impact of URRMI on the health utilization of urban and rural residents. We aimed to specify some recommendations on the policy of completely adopting the URRMI system in China, in addition to their practicability.

## 2 Materials and methods

### 2.1 Data source

**Figure 1:**
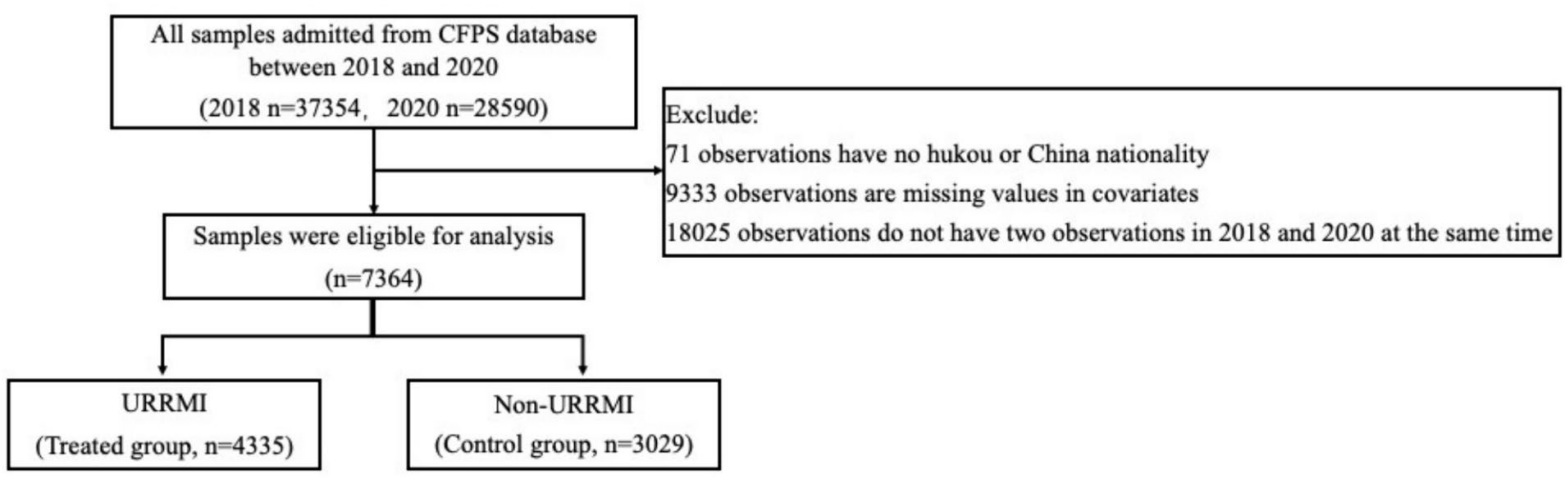
Study design and flow chart of the observations collection and the classification of observations with and without urban and rural residents medical insurance (URRMI) for propensity score matching. The n stands for the observations from 2018 and 2020, respectively. CFPS, China Family Panel Studies.

This study used data from the CFPS database, which was conducted in 2010 by the Chinese Peking University’s China Social Survey Center. The survey was organized into three sections: individual, family, and community, and it used stratified multi-stage sampling to collect data from 25 provincial administrative units. The poll covered various topics, including economic activity, educational success, family dynamics, population movement, and health. The information is reliable and true. Follow-up data of the CFPS database from 2018 and 2020 were used as experimental samples in this investigation. A final sample of 7,364 individuals was included in the regression after selection, and then separated into URRMI group (treated group) and non-URRMI group (control group) (Figure 1), including the sample data from the point where the two data periods intersected.

### 2.2 Model method

This study engrossed 1:1 k-nearest neighbor matching to determine how URRMI affected the self-rated health of Chinese individuals. A comparative experiment was designed to identify the unique effects of URRMI on individual self-assessment by comparing the self-rated health levels among the treated and control groups. This improved the hypothetical self-selection bias of the samples. When evaluating the health effects of URRMI on residents, it is crucial to account for selectivity bias based on observable variables like age, wealth, and family status produced by residents’ voluntary choice of insurance. We created a suitable control group using the PSM to avoid selectivity bias. PSM is frequently used to calculate the effects of health and other policy initiatives since randomized controlled testing is impractical (21).

The DID model was used for further analysis after resolving the problem of individual self-selection. Therefore, selectivity bias due to subjective observation and time-varying traits can be successfully mitigated using the variance. The difference between the average change in self-rated fitness levels in the experimental group from 2018 to 2020 was also compared based on PSM. Finally, the average treatment effect for the treated group (ATT) was obtained after eliminating selectivity bias from observable and unobservable characteristics.

### 2.3 Variables

#### 2.3.1 Explained variable

The explained variable of this study is self-rated health, which assesses the health level of the individual. It was coded as a two-valued dummy variable with a health status of unhealthy or average, defined as 0 = unhealthy, and a health status of relatively healthy, healthy, and very healthy, defined as 1 = healthy.

#### 2.3.2 Explanatory variables

The explanatory variable was the URRMI scheme. Since 2016, the URRMI Scheme has gradually amalgamated the URBMI and the NRCMS. Due to the questionnaire design of CFPS database, individuals who participated in URBMI or NRCMS were considered part of the URRMI for the 2018 statistics. Therefore, individuals who participated in URBMI, NRCMS, or URRMI were included in the URRMI for the 2020 statistics. People who did not purchase URRMI in 2018 and those who purchased URRMI in 2020 were included in the treated group. The control group consisted of people who did not purchase URRMI in both 2018 and 2020, as reflected by the grouping dummy variable treated (Table 1). However, because this study’s model is a double difference model, the product of the interactive term of the time and the treated group dummy variables (Time*Treated) was the primary explanatory variable to effectively quantify the impact of urban and rural basic medical insurance on the health of residents.

**Table 1:** Grouping information for the treated group and control group in 2018/2020.

	2018	2020
treated group	not purchase URRMI (included URBMI or NRCMS)	purchase URRMI (included URBMI, NRCMS, or URRMI)
control group	not purchase URRMI (included URBMI or NRCMS)	not purchase URRMI (included URBMI, NRCMS, or URRMI)

#### 2.3.3 Covariates

In this study, we used the Grossman model (22) as a template and selected three levels of control variables based on relevant literature (23–25): (1) Individual characteristics: Hukou status, Age, Gender, Marital status, Highest education, Annual income; (2) Selection of medical and health services: Hospitalization, Medical expenditure, Hospital scale, Clinic satisfaction, Hospital quality; and (3) Health behavior: Smoking, Drinking, and Napping.

Regarding individual characteristics, Hukou status was coded as 1 = rural hukou and 0 = urban hukou. Age and Gender were not treated, based on the original. Marital status was defined by the presence or absence of a spouse. Individuals who were married or cohabiting were considered to have a spouse, while those who were unmarried, divorced, or widowed were defined as not having a spouse. The variable Highest education was treated similarly, retaining the individuals who had primary education, junior secondary education, or senior secondary education. Individuals who were not in school and illiterate/semi-literate were merged into the illiterate/semi-literate category. Additionally, junior college, undergraduate degree, master’s degree, and doctorate were classified as higher education. The observed values of Annual income were log-transformed, basing on the actual income data. Regarding medical and health services, Hospitalization, Hospital scale, Clinic satisfaction, and Hospital quality were not processed, while Medical expenditure was log-transformed. According to the questionnaire, Hospitalization is a binary question, whereas it also incorporates five categories of Hospital scale and three different attitudes toward Clinic satisfaction. Regarding health behaviors, Smoke status was defined as smoking, which included quitting smoking. Drink status and Nap were not treated. Following the questionnaire, we have defined Drink status as indicating whether an individual has consumed alcohol three times a week in the past month, and Nap as indicating whether an individual has taken the nap. The assignment and definition of each variable are outlined in Table 2.

**Table 2:** Variable assignment of covariates.

Variables	Variable assignment
Individual characteristics	
Hukou status	1 = rural hukou 0 = urban hukou
Age	the actual age of the samples
Gender	1 = male 0 = female
Marital status	1 = married 0 = unmarried
Highest education	0 = illiterate/semi-literate category 1 = primary education 2 = junior secondary education 3 = senior secondary education 4 = higher education
Annual income	the actual annual income of samples with log-transformed
Selection of medical and health services	
Hospitalization	1 = yes 0 = no
Medical expenditure	actual medical expenditure of samples with log-transformed
Hospital scale	1 = general hospital 2 = specialist hospital 3 = community healthcare center/township hospital 4 = community healthcare clinic/village clinic 5 = clinic
Clinic satisfaction	1 = excellent 2 = very good 3 = good 4 = poor 5 = disappointed
Hospital quality	1 = excellent 2 = very good 3 = good 4 = poor 5 = disappointed
Health behavior	
Smoke	1 = yes 0 = no
Drink	1 = yes 0 = no
Nap	1 = yes 0 = no

#### 2.3.4 Statistical analysis

This study started with PSM therapy. The projected likelihood of each sample participating in URRMI was calculated using treated as the explanatory variable, a logit model for regression was built, and a new set of observation samples were created by matching using the 1:1 nearest neighbor matching approach. The model settings are shown in Equation 1:

(1) $$logit\left(Treated_{i}=1\right)=\gamma_{0}+\gamma_{1}X_{i}+\varepsilon_{it}$$

where *i* represents the individual. *Treated* indicated participation in URRMI and was assigned a value of 1 if the individual participated and 0 otherwise. *X_i_* is the matching variable, which included Hukou status, Gender, Marital status, Annual income, Hospitalization, Medical expenditure, Hospital scale, Clinic satisfaction, Hospital quality, Smoke, Drink, and Nap. *ε_it_* expresses error terms and contains information other than the main variables of the model. The current study aims to identify, for each individual in the treated group who purchased URRMI, a matched control individual in the same year who did not secure URRMI, and to eliminate samples that were not magnificently matched. The matched statistics will then be composed year by year and a DID regression will be presented on the combination of general statistics.

The following equation of the DID model was estimated for continuous outcomes:

(2) $$\begin{array}{l} Health_{it}=\alpha_{0}+\alpha_{1}Treated_{it}+\alpha_{2}Time_{it}+\\ \alpha_{3}\left(Treated_{it}\times Time_{it}\right)+\beta_{i}\times X_{it}+\varepsilon_{it} \end{array}$$

Where *Health_it_* stands for self-rated health of resident *i* at time *t*; the treated variable *Treated_it_* is a binary indicator; it epitomizes the group dummy variable. *Treated_it_* = 1 indicates that an individual *i* belongs to the treated group, having participated in the URRMI. *Treated_it_* = 0 epitomizes the control group, indicating that the individual *i* did not participate in the URRMI. *Time_it_* epitomizes the time dummy variable, with *Time_it_* = 0 indicating the time *t* before the individual *i* participated in the URRMI (the year 2018) and *Time_it_* = 1 indicating the time *t* after the individual *i* participated in the URRM (the year 2020). The variable *Treated_it_ × Time_it_* signifies the collaboration among groups and time. *X_it_* epitomizes a set of individual covariates of individual *i* at time *t*. *ε_it_* expresses error terms and contains information other than the main variables of the model.

Stata 15.0 in Mac was used for statistical cleaning analysis. The two-sided statistical significance level was set to 0.05.

## 3 Results

### 3.1 Samples characteristics

After statistic cleaning and PSM matching, we analyzed a total of 2,021 treated group samples and 1,372 control group samples from 2018, as well as 2,314 treated group samples and 1,657 control group samples from 2020 (Table 3). The overall self-rated health of the samples was good, with each average health status above 0.78, 0.81, 0.75, and 0.80. Comparing the standard deviations of the four groups, it can be seen that the differences among the four groups are not significant (standard deviation [SD] 0.42, 0.39, 0.43, and 0.40, respectively). The average Hukou status of both two treated groups was approximately 0.8, concentrated in rural regions. In contrast, the average Hukou status of both two control groups was less than 0.5, with the majority of individuals living in urban regions. The average age of the 2018 treated group was 41.25 years (SD 12.83), while the average age of the 2018 control group was 38.33 years (SD11.35). Similarly, the average age of the 2020 treated group was 40.23 years (SD12.62), and the average age of the 2020 control group was 38.6 years (SD11.42). The gender distribution was relatively balanced within each group, and the differences between groups were not significant. The average Marital status of all four groups was above 0.5, indicating that the majority of participants were married. The average for the Highest education was around 1.3, indicating that the majority of individuals had completed compulsory education. Similarly, there were no significant differences in Annual income between the four groups. The average Hospitalization score for each group was 0.12, 0.1, 0.12, and 0.1, respectively (SD 0.32, 0.29, 0.32, and 0.3, respectively), indicating that the majority of participants had not been hospitalized due to illness. The average Hospital scale for each group was 2.95, 2.32, 3.09, and 2.29, respectively (SD 1.63, 1.62, 1.62, and 1.61, respectively), indicating that the majority of participants had been treated at specialist hospitals or community health centers. The average Clinic satisfaction was between general and satisfied. The average Hospital quality was between general and good. The average Smoke for each group was above 0.5, with the 2018 control group having a score of 0.63, indicating that smoking behavior was prevalent. The average of Drink for each group was around 0.1, indicating that drinking behavior was less common. The average Nap score for each group was around 0.6, with the 2020 experimental group being close to 0.5, indicating that the differences between individuals who habitually take naps and those who do were not significant, but there were slightly more individuals who did not habitually take naps.

**Table 3:** Characteristics of the study sample in the CFPS 2018 and 2020.

Variables	2018				2020			
	Treated		Control		Treated		Control	
	(*n* = 2,021)		(*n* = 1,372)		(*n* = 2,314)		(*n* = 1,657)	
	Mean	SD	Mean	SD	Mean	SD	Mean	SD
Health	0.78	0.42	0.81	0.39	0.75	0.43	0.80	0.40
Individual characteristics								
Hukou status	0.77	0.42	0.41	0.49	0.82	0.39	0.37	0.48
Age	41.25	12.83	38.33	11.35	40.23	12.62	38.60	11.42
Gender	0.56	0.50	0.55	0.50	0.57	0.50	0.56	0.50
Marital status	0.79	0.41	0.75	0.44	0.83	0.37	0.78	0.42
Highest education	1.36	1.20	2.53	1.30	1.20	1.07	2.47	1.29
Annual income	10.09	1.03	10.71	0.91	9.98	0.95	10.53	0.82
Selection of medical and health services								
Hospitalization	0.12	0.32	0.10	0.29	0.12	0.32	0.10	0.30
Medical expenditure	7.24	1.67	7.27	1.61	7.20	1.57	7.26	1.53
Hospital scale	2.95	1.63	2.32	1.62	3.09	1.62	2.29	1.61
Clinic satisfaction	3.72	0.77	3.74	0.74	3.56	0.82	3.58	0.80
Hospital quality	3.56	0.91	3.64	0.86	3.39	0.86	3.46	0.84
Health behavior								
Smoke	0.59	0.49	0.63	0.48	0.54	0.50	0.59	0.49
Drink	0.14	0.35	0.13	0.33	0.16	0.37	0.15	0.36
Nap	0.62	0.49	0.65	0.48	0.51	0.50	0.58	0.49

### 3.2 Balancing proper test for PSM results

The aforementioned model of Equation 1 was used to determine PSM. The balancing characteristics of the observable covariates were assessed between the treated groups and control groups using k-nearest neighbor matching to reduce sampling bias (Table 4). Unmatched refers to the treated group and control group samples before matching, whereas matched refers to the treated group and control group samples with approximately constant distribution following propensity matching.

**Table 4:** Balance test for propensity score matching.

Variable	Unmatched	Mean		%bias	%reduc-tion	*t*-test	
	Matched	Treated	Control			*t*	*p* > *t*
Individual characteristics							
Hukou status	U	0.796	0.387	91.400		39.250	0.000
	M	0.796	0.798	−0.600	99.400	−0.290	0.769
Gender	U	0.561	0.554	1.400		0.590	0.555
	M	0.562	0.554	1.500	−9.900	0.710	0.475
Marital status	U	0.814	0.763	12.500		5.330	0.000
	M	0.814	0.769	11.000	12.100	5.140	0.000
Annual income	U	10.035	10.614	−62.100		−25.930	0.000
	M	10.045	10.012	3.600	94.200	1.550	0.122
Selection of medical and health services							
Hospitalization	U	0.116	0.099	5.400		2.260	0.024
	M	0.116	0.105	3.700	32.000	1.680	0.093
Medical expenditure	U	7.221	7.263	−2.700		−1.120	0.263
	M	7.220	7.187	2.100	20.800	0.970	0.334
Hospital scale	U	3.020	2.303	44.300		18.690	0.000
	M	3.020	3.059	−2.400	94.600	−1.100	0.273
Satisfaction with medical conditions	U	3.632	3.650	−2.300		−0.970	0.331
	M	3.632	3.628	0.500	79.700	0.210	0.830
Medical level	U	3.467	3.540	−8.400		−3.550	0.000
	M	3.467	3.500	−3.700	55.800	−1.700	0.090
Health behavior							
Smoke	U	0.563	0.610	−9.700		−4.100	0.000
	M	0.562	0.576	−2.800	71.500	−1.280	0.200
Drink	U	0.152	0.139	3.800		1.600	0.111
	M	0.152	0.149	0.900	77.500	0.390	0.696
Nap	U	0.557	0.610	−10.700		−4.490	0.000
	M	0.557	0.549	1.800	83.300	0.820	0.411

**Figure 2:**
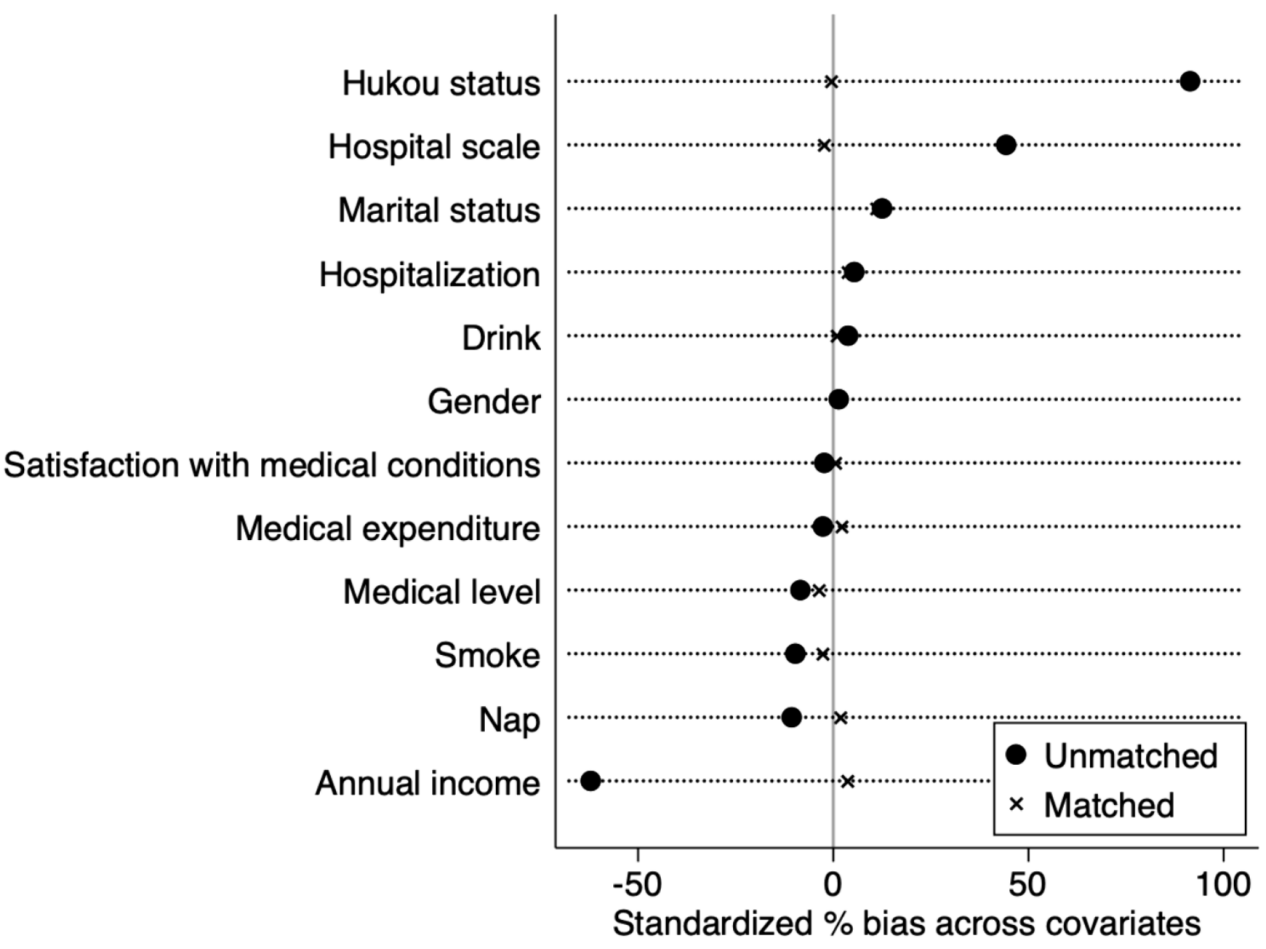
Balance test results of tendency propensity score matching score.

Age, Medical level, Smoke, and Nap were significantly different before and after matching. Rosenbaum argues that the matching result is reliable when the absolute value of the matching variable’s standard deviation is less than 20% (26). Table 4 shows that, except for age and education, the standard bias of all variables after matching was within 20%, indicating that the matching result was credible. The visualization of the matching results is shown in Figure 2. Generally, using the PSM efficiently minimized discrepancies in numerous individual characteristics among the treated group and control groups before sample matching, and the treated group and control groups were more reliable.

### 3.3 Effect

The influence of URRMI on self-rated health of inhabitants was calculated using k-nearest neighbor matching. Table 5 shows the results of PSM-DID regression on randomly selected samples. Treated ×Time, hukou status, age, highest education, annual income, medical expenditure, hospital scale, clinic satisfaction, and napping were found to have an impact on the self-rated health of residents. The regression coefficient of the interaction term Treated ×Time was 0.456 and was statistically significant at the 10% level. This means that those who opted to engage in URRMI had a 0.456-unit greater coincidental in their self-rated health improvement compared to those who did not. Evidently, URRMI has a substantial impact on people’s health.

**Table 5:** Propensity score matching and difference-in-difference result graph (Health).

Health	Coef.	Std. Err.	*z*	*P* > z	[95% Conf.]	[Interval]
Treated ×Time	0.456	0.257	1.770	0.076	−0.048	0.960
Time	−0.238	0.183	−1.300	0.195	−0.597	0.122
Treated	−0.252	0.183	−1.070	0.169	−0.611	0.106
Individual characteristics						
Hukou status	0.311	0.149	2.080	0.037	−0.604	−0.019
Age	−0.046	0.008	−6.020	0.000	0.031	0.062
Gender	0.036	0.194	0.190	0.852	−0.416	0.344
Marital status	−0.034	0.185	−0.180	0.853	−0.329	0.398
Highest education	0.256	0.069	3.680	0.000	−0.391	−0.120
Annual income	0.162	0.080	2.030	0.043	−0.318	−0.005
Selection of medical and health services						
Hospitalization	0.271	0.224	1.210	0.227	−0.710	0.168
Medical expenditure	−0.464	0.061	−7.650	0.000	0.345	0.583
Hospital scale	0.106	0.044	2.400	0.016	−0.193	−0.020
Clinic satisfaction	0.427	0.101	4.220	0.000	−0.626	−0.229
Hospital quality	0.050	0.090	0.560	0.577	−0.226	0.126
Health behavior						
Smoke	−0.077	0.191	−0.400	0.688	−0.298	0.452
Drink	0.295	0.200	1.480	0.140	−0.686	0.097
Nap	0.232	0.136	1.710	0.088	−0.498	0.035
_cons	2.918	1.038	2.540	0.005	−4.750	−0.611

Hukou status also had an impact on the self-rated health of residents. The regression coefficient of the PSM-DID was 0.311 and was significant at the statistical level of 5%, indicating that the self-rated health of rural hukou had a superior chance of ameliorating to urban hukou. Age also influenced the self-rated health of residents, and the regression coefficient for the self-rated health of residents was −0.046, which was significant at the statistical threshold of 1%, indicating that younger residents had the preferable probability of self-rated health. Contrary to traditional ideas, older individuals felt that their bodies were unhealthy. The regression coefficient for the variable highest education was 0.256 and statistically significant at the 1% level, showing that the higher the educational background, the better likelihood of the self-rated health. In contrast, individuals with a high educational background rated their health status as satisfactory. Annual income also had a significant impact on the self-rated health of residents at the 5% statistical level, with a regression coefficient of 0.162, indicating that a higher annual income had more possibility for self-rated health and vice versa. Medical expenditure had a statistically significant effect on the self-rated health of residents at the 1% statistical level, and the regression coefficient was −0.464, indicating that payment of more medical expenses modified the chances of enhancing self-rated health while paying more medical expenditure worsened self-rated health. The Hospital scale statistically significantly affected residents’ self-rated health at the 5% level. The regression coefficient was 0.106, indicating that individuals who go to major hospitals perceived that their health was better, but this observation may be a problem of two-way choice, which needs further research. In the CFPS questionnaire, medical conditions include medical equipment, drugs, medical quality, hospitalization conditions, the distance to seek medical treatment, and the convenience of transportation. Patients strived for advanced medical care at hospitals of different scales and provided their satisfaction ratings on the medical services they received. Through the statistics, we found clinic satisfaction had a statistically significant effect on the self-rated health of residents at the statistical level of 1%, and the regression coefficient was 0.427, indicating that residents who were satisfied with the hospital services were more likely to have the finest health condition. Nap had a significant influence self-rated health of residents at the statistical level of 10%. The regression coefficient was 0.232, indicating that individuals who has lunch break habits were more potential to possess favorable self-rated health. The study found that the URRMI had a significant effect on improving the health status of insured individuals. Insured individuals had a higher probability of having better health status compared to those who did not purchase insurance. Younger individuals, those with higher education levels, those with higher average annual income, those who spent less on medical expenses, larger scale of medical treatment, and higher satisfaction with medical treatment, those who has a nap habit had a higher probability of improvement in health status.

### 3.4 Further exploration of URRMI and medical expenditure

To delve deeper into the factors that influence URRMI in objective health indicators, we have devised a novel model that examines the correlation between URRMI and medical expenses. This model offers a more thorough understanding of the interplay between URRMI and self-perceived health status of individuals. Specifically, we have introduced a new dependent variable, expense. This variable is assigned a value of 1 if the Medical expenditure of a given sample constitutes less than 5% of its annual income; otherwise, it is assigned a value of 0. As for the explanatory variables and covariates, we have eliminated the original Medical expenditure variable while retaining all other variables unchanged.

In conducting this analysis, we continue to utilize the PSM-DID model that was previously mentioned, as it remains a suitable framework for examining the relationships and effects in question. The model settings are shown in Equation 3:

(3) $$logit\left(Treated_{i}=1\right)=\gamma_{2}+\gamma_{3}X_{i}+\varepsilon_{it}$$

where *i* represents the individual. *Treated* indicated participation in URRMI and was assigned a value of 1 if the individual participated and 0 otherwise. *X_i_* is the matching variable, which included Hukou status, Gender, Marital status, Annual income, Hospitalization, Hospital scale, Clinic satisfaction, Hospital quality, Smoke, Drink, and Nap. *ε_it_* expresses error terms and contains information other than the main variables of the model.

The following equation of the DID model was estimated for continuous outcomes:

(4) $$\begin{array}{l} expense_{it}=\alpha_{4}+\alpha_{5}Treated_{it}+\alpha_{6}Time_{it}+\\ \alpha_{7}\left(Treated_{it}\times Time_{it}\right)+\beta_{i}\times X_{it}+\varepsilon_{it} \end{array}$$

Where *expense_it_* stands for the expenditure of medical *i* at time *t*, with Medical expenditure ≤ 5%Annual income = 1, Medical expenditure >5%Annual income = 0. The treated variable *Treated_it_* is a binary indicator; it epitomizes the group dummy variable. *Treated_it_* = 1 indicates that an individual *i* belongs to the treated group, having participated in the URRMI. *Treated_it_* = 0 epitomizes the control group, indicating that the individual *i* did not participate in the URRMI. *Time_it_* epitomizes the time dummy variable, with *Time_it_* = 0 indicating the time *t* before the individual *i* participated in the URRMI (the year 2018) and *Time_it_* = 1 indicating the time *t* after the individual *i* participated in the URRM (the year 2020). The variable *Treated_it_ × Time_it_* signifies the collaboration among groups and time. *X_it_* epitomizes a set of individual covariates of individual *i* at time *t*. *ε_it_* expresses error terms and contains information other than the main variables of the model.

Table 6 presents the results of a PSM-DID analysis of the dependent variable, expense, and its independent variables. We can observe that the coefficient for the key variable Treated × Time is −0.357, which is significant at the 5% level. This indicates that for the treated group who purchased insurance, their likelihood of incurring higher medical expenses has increased. Compared to the control group who did not purchase insurance, the treated group who did purchase insurance has a higher probability of spending more on medical expenses. This may suggest that the insurance purchase behavior has, to some extent, increased the utilization of medical insurance. This is also the outcome we anticipated.

**Table 6:** Propensity score matching and difference-in-difference result graph (expense).

Expense	Coef.	Std. Err.	*z*	*P* > z	[95% Conf.]	[Interval]
Treated ×Time	−0.357	0.155	−2.31	0.021	−0.660	−0.054
Time	0.154	0.121	1.27	0.205	−0.084	0.392
Treated	0.184	0.116	1.59	0.113	−0.043	0.410
Individual characteristics						
Hukou status	0.096	0.097	0.99	0.321	−0.094	0.286
Age	−0.026	0.004	−6.62	0.000	−0.034	−0.018
Gender	0.507	0.119	4.28	0.000	0.275	0.740
Marital status	−0.060	0.108	−0.55	0.580	−0.272	0.152
Highest education	0.238	0.041	5.85	0.000	0.158	0.318
Annual income	1.242	0.063	19.79	0.000	1.119	1.364
Selection of medical and health services						
Hospitalization	−4.031	0.199	−20.30	0.000	−4.420	−3.642
Hospital scale	0.327	0.027	12.10	0.000	0.274	0.380
Clinic satisfaction	0.173	0.058	2.99	0.003	0.060	0.286
Hospital quality	−0.136	0.053	−2.53	0.011	−0.240	−0.031
Health behavior						
Smoke	0.024	0.119	0.21	0.837	−0.209	0.257
Drink	0.244	0.119	2.05	0.041	0.010	0.478
Nap	0.089	0.079	1.13	0.259	−0.065	0.243
_cons	−12.005	0.696	−17.25	0.000	−13.370	−10.641

### 3.5 Testing the robustness of PSM-Did estimation

In this study, a placebo test was used as the robustness verification method. The specific implementation method involved randomly selecting the same number of samples as the original control group as the dummy control group from the samples. The remaining samples were then designated as the dummy experimental group. The regression parameters were re-estimated 500 times under the condition, keeping the relevant control variables, matching methods, and regression steps unchanged.

**Figure 3:**
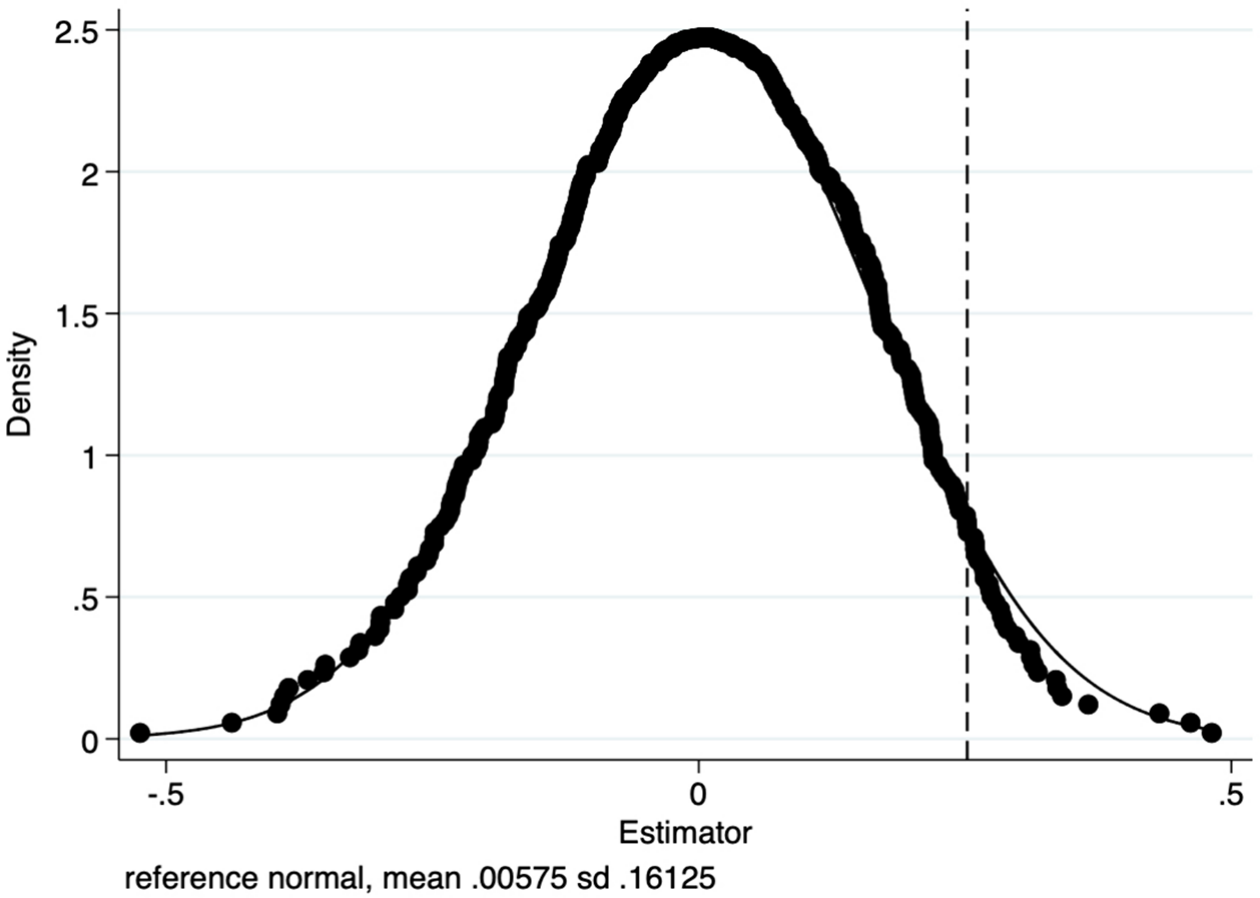
K-Nearest neighbor matching the distribution of 500 random sampling regression coefficients.

A placebo test was used to validate the reliability of the PSM-DID approach for estimating the core explanatory variable α *_3_*. Figure 3 depicts the distribution of estimated coefficients for the interaction term after 500 trials, with the estimated coefficients shown. Close to the ordinary normal distribution, α *_3_* was evenly dispersed about 0. The position of coefficient α *_3_* (0.337) is indicated by the vertical bar (Figure 3). This number emerges at the end of the kernel density distribution plot, demonstrating that basic medical insurance does not significantly improve the health of insured residents under the URRMI scheme once bogus experiments are constructed. This thus lends credence to the preceding conclusions.

## 4 Discussion

The PSM-DID methodology effectively combines the strengths of PSM and DID analysis. Specifically, PSM aids in selecting control group who possess similar characteristics to the treated group, thus mitigating the potential for endogenous self-selection bias. Conversely, DID serves to precisely quantify the impact of the URRMI policy by comparing the changes observed in the treated group with those in the control group, both before and after the implementation of the URRMI policy. There is ample evidence from numerous journal articles to support that this model is widely utilized in the medical domain as well as in other disciplines due to its robust analytical framework. A study adopted PSM-DID to evaluate whether the aggregated payment based on cases of acute patients and chronic patients is more cost-effective than the traditional payment of a daily allowance for acute and chronic patients (27). A Japanese study examined the spillover consequence of the Japanese public long-term care insurance (LTCI) as a policy to encourage labor force participation of family caregivers from 1995 to 2013 (28). The study examined the spillover consequence in two periods: before and after the LTCI’s introduction in 2000 and before and after its major amendment in 2006 (28). A study from China used PSM-DID to assess the impact of the URRMI scheme on how often urban and rural residents in the four experimental areas used medical services (29). Using panel data, a different study used PSM-DID to examine the targeting and impact of the Askeskin program, a sponsored social health insurance program for the underprivileged and those living in poverty. The study showed that the program targeted the underprivileged and those who are most at risk of catastrophic out-of-pocket medical expenses (30). A study used PSM-DID to assess the impact of the restructurings on the two most prevalent childhood infections, the incidence and treatment of fever (malaria) and diarrhea infections, after introducing the national health insurance scheme restructurings (31). Notably, previous evaluation studies have not examined these outcomes after introducing the national health insurance scheme reforms (31). To assess the impact of China’s National Essential Medicine Policy (NEMP) on outpatient treatment utilization and spending in Tianjin, China (32). Accordingly, the utilization of this robust PSM-DID methodology in this study not only adeptly mitigates the challenges posed by endogenous self-selection bias but also offers a rigorous analysis and insightful discussion of the causal relationship between the URRMI policy and self-rated health outcomes.

This study investigated the impact of URRMI on the health of residents and showed that URRMI can greatly improve the health of insured people. Like most prior conclusions, this study indicates that URRMI has a considerably favorable impact on the health of inhabitants. This could be because enrolling in health insurance can affect older persons’ decisions to seek medical care, thereby dramatically reducing healthcare demand (HCD) and healthcare expenditure (HCE) (33). URRMI reduces out-of-pocket expenditure for residents, relieves the financial burden of medical expenses, and lowers residents’ medical costs. It also gives people a sense of security and solves the most fundamental health and livelihood issues. According to some studies, there is a time lag between the recovery and improvement of residents’ health levels after enrolling in basic medical insurance. This implies that the change in residents’ health levels caused by the accumulation of health stock is quantitative to the qualitative change process. This makes measuring the influence of URRMI on residents’ health at the start of implementation or over a short period difficult (34). The participation rate of basic medical insurance in China has remained above 95% throughout the year, but it has not achieved full coverage. Referring to the reform of the medical insurance system in Germany and Japan, the main way to quickly realize universal medical insurance is to force residents to participate in insurance by legislation. Therefore, the modification of URRMI in China needs to be coordinated with the corresponding laws and regulations to further improve the coverage of the scheme. In this way, the law can be a factor in amplifying the health performance of basic medical insurance.

Compared with objective health indicators, choosing self-rated health status as the dependent variable of this study has many advantages. Firstly, it reflects individuals’ subjective perception of their own health status, which can better reflect individuals’ true feelings than objective indicators. Secondly, self-rated health status is easy to collect and does not require complex medical examinations or biological indicator measurements, reducing research costs and difficulties. Lastly, self-rated health status reflects non-biological factors such as individuals’ psychological state and lifestyle, which often have important impacts on health status and can be examined through self-rated health status. However, self-rated health status is prone to subjective factors, and its evaluation may differ among individuals based on their psychological state, social and cultural background, and health knowledge level. Additionally, it does not reflect biological information that objective indicators, such as blood pressure and blood sugar, can indicate, which are important for evaluating the health status of the individual.

We also found that the geographic difference between urban and rural hukou has a significant effect on the health of residents. Our statistical results show that rural hukou had better self-rated health status. Firstly, the household registration system is a unique administrative system of identity management in China. The state lawfully collects, confirms, and registers basic information such as birth, death, kinship, and address of residents, to safeguard the rights and interests of some residents in employment, education, social welfare, and other aspects. Therefore, it has also formed a dual structure of urban and rural areas specific to the context of China. As the results illustration, the characteristics presented by urban hukou and rural hukou are noticeably distinctive. Additionally, Fang H et al. emphasized in their article that urban inhabitants consistently revelation poorer health outcomes compared to rural residents, with a sensitive risk of being diagnosed with critical illnesses (35). The unambiguous contrast in the natural environment and lifestyle between urban and rural zones is noteworthy. The rural environment is relatively fresher, without continuous exhaust pollution, light pollution, and noise pollution. Despite the ongoing efforts to enhance the construction of green areas in urban area, it is incontrovertible that rural areas possess a more abundant presence of green spaces. Notably, a study has authenticated the momentous role of green spaces in optimistically enhancing self-perceived general health and mental well-being, thus corroborating our findings (36). Thirdly, the gradual expansion of medical resources into rural zones has led to substantial improvements in the rural healthcare system and its resources (37). This has translated into a significant enhancement in basic medical service capabilities, surpassing previous standards. The rural health education and promotion initiatives are being undertaken on an extensive scale. Through the placement of health promotion posters and the organization of public welfare lectures at village activity centers, the health literacy and healthy behavior patterns of rural residents have been strengthened. Such endeavors are instrumental in preventing and managing the prevalence of various chronic diseases. However, the adjustment of urban and rural medical insurance to urban resident medical insurance has changed the medical insurance benefits, which helps to narrow the urban–rural gap to some extent. After combating poverty, the government has also implemented multiple policies to ensure the effective linkage between consolidating and expanding the achievements of poverty alleviation and promoting rural revitalization. The reform of the medical insurance system will be continuously intensified to ensure basic medical security for low-income rural residents and firmly prevent a large-scale return to poverty caused by illnesses. Further research and continuous monitoring may be needed to assess the impact of the changes caused by the deepening reform of the medical security system.

Given the differences among urban and rural zones, the reform path of URRMI can start from the following three aspects. First, the government must persist in enhancing the rural grass-roots medical system. It is highly recommended that the government annually augment its financial investment in rural grass-roots medical services and facilities by a designated percentage, thereby guaranteeing a steady increase in medical resources in rural areas. Regarding resource allocation, the government must effectively ensure the efficient and rational utilization of resources, tailored to the specific needs and population structure of each region. Additionally, incentives should be offered to medical graduates to work in grass-roots medical institutions, accompanied by commensurate policy support and welfare benefits. Second, the government should further intensify the reform of the URRMI payment plan and promote a diversified composite medical insurance payment method (38), aiming to better accommodate the diverse characteristics of medical services. Third, improve the multi-level construction process of URRMI systems, divide rural areas into different medical insurance access standards through financial subsidies, and regulate the process of social wealth redistribution. Fourth, the reform of the URRMI should align with the government’s macro-control measures on the medical and health service market to improve the influence of purchasing with quantity on the prices of medical drugs and devices. Fifth, government agencies encompassing health, education, and culture ought to intensify their interdepartmental collaboration and harness the power of diverse social media platforms to disseminate information on health literacy and policies pertaining to basic medical insurance for both urban and rural residents, ultimately fostering a deeper understanding and awareness among the populace.

Contrary to previous research findings, our study presents a novel discovery: traditional health behaviors such as smoking, alcohol consumption, and napping do not always have absolute and unchanging effects on health. Specifically, our data indicates that simply controlling smoking and drinking habits does not significantly improve health outcomes. However, it is noteworthy that we found that appropriate napping has shown significant positive impacts on health and regression analysis, strongly suggesting that individuals with a habit of napping tend to have better health conditions. This conclusion aligns with numerous mainstream studies. Among various research reports, one study particularly caught our attention. It suggests that brief afternoon naps indeed have a positive effect on promoting human health (39). This discovery further reinforces our view that good health habits are of indispensable importance for maintaining individual health status.

After conducting a further exploration of URRMI and Medical expenditure, we discerned a notable rise in the probability of the experimental group encountering steeper medical costs compared to other groups. Moreover, our analysis of self-rated health levels revealed a compelling pattern: as medical expenses escalated among the sampled individuals, their self-assessed health status showed a concurrent decline. These findings align closely with previous research, indicating a correlation between increased health service utilization and a decline in self-rated health (40). This pattern suggests that medical insurance purchases, to a degree, have encouraged the utilization of medical insurance benefits, yet it is also intertwined with elevated medical necessities and health challenges. Evidently, URRMI shifts the burden of personal medical expenses onto the insurance system, prompting insured individuals to seek out specialized medical services and facilities whenever illness strikes. This augmentation in healthcare utilization is inevitably mirrored in the utilization of medical insurance. Nevertheless, this trend may also stem from heightened medical needs and health issues. Those who frequently avail themselves of healthcare services often rate their health status lower, likely due to the more significant health problems they encounter, necessitating more extensive medical interventions. Consequently, when delving into the intricate relationship between medical insurance purchases, medical expenditures, and self-rated health, we must adopt a multifaceted approach, considering various factors. Future research can delve deeper into the interplay between these variables and explore ways to refine medical insurance policies to enhance overall health standards and quality of life.

## 5 Limitations

This study had respective limitations. First, a significant assumption of PSM-DID regression is that the model should contain all the covariates before and after the match that may affect the effectiveness of the strategy. Unobservable covariates will cause divergent tendencies between treated groups and control groups, which may bias the results of this study. Second, PSM is for section data, and DID is for panel data. Generally, there are two solutions to solve the problem of different application ranges. Panel data can be converted into section data for processing, and then phase-by-phase matching is conducted on each phase section of panel data. This study adopted the phase-by-phase matching method of a balanced panel. Although this method can solve the problem of sample matching in different periods, special class variables might still cause sample mismatch (41). Finally, the survey relied on specific time points and did not continue to study and compare data before 2018. Consequently, the data from the two periods are not significant in the short term, and the long-term effects of policies are ignored.

## 6 Conclusion

This study further confirms that PSM-DID is an imperative tool for scrutinizing the influence of the amalgamation of URRMI on self-rated health status through 2 years of panel data. Moreover, the statistics imply that purchasing in URRMI has an optimistic impact on the health of residents, promoting better self-rated health performance. In addition, we also institute regional differences in health, with urban residents having superior health status compared to rural residents. Furthermore, our study challenges previous research, revealing that traditional health behaviors have varying effects, with smoking and drinking control not significantly improving health, while appropriate napping positively impacts health, aligning with mainstream studies and emphasizing the importance of good health habits. Our further exploration of URRMI and medical expenditure revealed a rise in medical costs for the experimental group and a correlation between increasing expenses and declining self-rated health, aligning with research showing a link between increased health service use and poorer self-rated health.

We recommend that the government expand the coverage of the URRMI scheme, narrow the gap among urban and rural zones, and improve the implementation of certain insurance subsidies and benefits for rural hukou to improve the overall health level of residents. Furthermore, the government can support medical institutions to provide better and more affordable medical facilities for urban and rural residents by raising the standard and scope of medical insurance payments. Finally, policymakers should learn from the implementation of the experimental areas, improve the laws and regulations related to URRMI, establish a sound medical insurance supervision mechanism, strengthen the administration and supervision of medical insurance funds, and formulate more efficient implementation strategies for the forthcoming nationwide promotion.

## Data availability statement

The original contributions presented in the study are included in the article/supplementary material, further inquiries can be directed to the corresponding author.

## Author contributions

YS-Y: Conceptualization, Data curation, Formal analysis,Investigation, Methodology, Writing – original draft, Writing – review & editing. CY-T: Writing – review & editing. XX-Y: Writing – review & editing. WD: Writing – review & editing. LX-H: Writing – review & editing. LW: Writing – review & editing. PT: Writing –review & editing. MX-H: Conceptualization, Writing – review & editing.
